# **A randomized controlled trial demonstrating sustained benefit of autologous matrix-induced chondrogenesis (AMIC** ^®^ **) over microfracture: 10-year follow-up**

**DOI:** 10.1007/s00590-024-03948-0

**Published:** 2024-04-17

**Authors:** Martin Volz, Jens Schaumburger, Jörg Gellißen, Joachim Grifka, Sven Anders

**Affiliations:** 1 Sportklinik Ravensburg, Ravensburg, Germany; 2 Department of Orthopedic Surgery, Asklepios Clinical Center Bad Abbach, University of Regensburg, Kaiser-Karl V.-Allee 3, 93077 Bad Abbach, Germany; 3 Radiologische Allianz, Hamburg, Germany

**Keywords:** RCT, Chondral, Knee, Cartilage, Repair, Microfracture, AMIC^®^

## Abstract

**Purpose:**

Autologous matrix-induced chondrogenesis (AMIC^®^) and microfracture are established treatments for focal chondral defects in the knee, but there are little clinical data concerning these procedures over the long term. This study evaluates the outcomes of AMIC^®^ compared to microfracture over 10-year follow-up.

**Methods:**

Forty-seven patients were randomized and treated either with MFx (*n* = 13), sutured AMIC^®^ (*n* = 17) or glued AMIC^®^ (*n* = 17) in a prospective, randomized, controlled multicentre trial. The Modified Cincinnati Knee Score, a visual analogue scale for pain and MOCART score were used to assess outcomes over 10 years post-operatively.

**Results:**

All treatment arms improved in the first 2 years, but a progressive and significant deterioration in scores was observed in the MFx group, while both AMIC^®^ groups remained stable. MOCART scores were comparable between groups.

**Conclusion:**

The AMIC^®^ procedure results in improved patient outcomes in comparison with microfracture up to 10 years following surgery for the repair of focal chondral defects in the knee.

*ClinicalTrials.gov Identifier:* NCT02993510

## Introduction

Surgery for focal knee cartilage lesions pursues the goal of chondral repair in order to restore full, pain-free joint function and preventing, or at least delaying, the early onset of osteoarthritis. In clinical practice, a plethora of surgical interventions aim to repair cartilage tissue and overcome the poor healing potential of articular cartilage. Understandably, a better quality of the repair tissue should result in a better long-term clinical outcome [1, 2].

Microfracture (MFx) perforates the subchondral bone in order to access the bone marrow compartment, releasing mesenchymal stem cells that can differentiate but are also immunoregulatory, serving to foster regenerative microenvironments in areas of tissue injury [3]. Therefore, a more comprehensive term for this is bone marrow stimulation (BMS) [4]. As a basic BMS procedure, MFx can provide good, initial results, but unfavourable long-term results have been reported, especially in larger lesions [5]. Considering this limitation, the combination of MFx with a collagen I/III scaffold, referred to as the autologous matrix-induced chondrogenesis (AMIC^®^) procedure, offers another prospective treatment option to overcome this burden. The bioresorbable membrane stabilizes the “super clot”, reduces edge loading of the surrounding cartilage and supports chondral differentiation by providing a biological chamber [6, 7] and therefore optimizes the conditions for successful cartilage repair, with the attendant clinical benefits. We therefore hypothesized that adding a collagen I/III scaffold onto a microfractured area in focal cartilage defects in the knee would result in superior outcome than with MFx alone.

Accordingly, we initiated a prospective, randomized controlled clinical trial (PRCT) to evaluate outcomes of both therapies. Short-term results had shown comparable improvements in pain and function [8]. These results, however, started to diverge by 5 years, with the MFx cohort displaying a worsening of pain and outcome scores in comparison with AMIC^®^-treated patient [9]. In continuation, the aim of this current study was to evaluate the 10-year outcomes of these cohorts.

## Material and methods

### Study design

This PRCT was designed to compare the efficacy and safety of the AMIC^®^ technique to MFx alone in the treatment of focal cartilage defects of the knee. Informed consent was given by every patient participating in this study. Enrolled patients were 18–50 years of age with one or two isolated, Outerbridge grade III or IV [10] cartilage defects of the knee and a defect size of 2–10 cm^2^. Exclusion factors, such as more than 2 defects, 2 corresponding defects or bilateral defects, signs of osteoarthritis, bone lesions deeper than 0.7 cm, axis deviation by clinical evaluation, unresolved knee instability, along with certain systemic diseases, were detailed in the previous publications [8, 9].

Patients were randomly assigned to one of 3 groups, receiving one of the following treatments: microfracture alone (MFx), sutured AMIC^®^ or glued AMIC^®^. Due to slow enrolment only 47 patients were available for evaluation in this study. The study design, comparing a total arthroscopic procedure (MFx alone) to an open procedure (AMIC^®^ glued or AMIC^®^ sutured), meant that neither patients nor physicians were blinded.

### Ethical approval

The study was approved by the Ethics Committee (03–088, 03/173-MZ and 20–1875-101, ZKS, University of Regensburg, Germany), conducted according to the Declaration of Helsinki and Good Clinical Practice and registered on clinicaltrials.gov (NCT02993510).

### Surgical technique

The surgical procedures and rehabilitation had been detailed in our previous publications [8, 9]. In summary, MFx was performed according to the technique published by Steadman et al. [11]. The surgical technique for the AMIC^®^ groups was performed using a mini-arthrotomy, as described in our initial paper concerning this cohort. Specifically, following the MFx procedure, a collagen type I/III matrix (Chondro-Gide^®^, Geistlich Pharma AG, Wolhusen, Switzerland) was added to cover the treated defect area. Chondro-Gide^®^ was placed with the porous layer facing the bone surface and fixed using either sutures (PDS 5.0, Ethicon, Norderstedt, Germany; sutured AMIC^®^) or by gluing the matrix onto the bone surface with fibrin glue (Tissucol, Baxter, Unterschleissheim, Germany; glued AMIC^®^). The stability of the matrix was checked by flexing and extending the knee 10 times. An intra-articular drain without suction was inserted, the wound was closed, and patients were hospitalized for 2–5 days after surgery.

### Post-operative rehabilitation program

All patients were assigned to the same rehabilitation protocol. The staged program included a progression of weight bearing over 6 weeks and mobilisation of the index knee and included electrotherapy of lower limb musculature, proprioceptive exercises and progression from walking to sports, as indicated by the patients’ clinical progression. Additionally, scar tissue management was part of the clinical routine.

### Clinical evaluation and data collection

Prior to surgery, all patients underwent physical examination and every patient had a standard X-ray of the knee and patella and MRI. All patients were followed prospectively.

Each patient was contacted and scheduled for clinical evaluation of the knee and collection of Patient Related Outcome Measures (PROMs) at 6 weeks, 3, 6, 12, 24, 60 and 120 months. MRI imaging was done at 6, 12, 24, 60 and 120 months. Any type of complication, injury or subsequent surgery was documented. Clinical outcome was assessed by the Modified Cincinnati score [12] and a visual analogue scale (VAS; 0–100) for pain, which ranged from 0 (no pain) to 100 (severe pain). Safety was evaluated by monitoring adverse events (AE). Radiological outcomes were assessed with magnetic resonance imaging (MRI, 1.5 T) by an independent and blinded radiologist using the MOCART score [13].

### Statistical analysis

Quantitative variables were described using the mean and standard deviation, whereas qualitative variables were reported with absolute and percentage frequencies. Cincinnati and VAS scores were analysed using the Brunner–Langer approach which is particularly suitable for the analysis of longitudinal data (repeated measures) with small sample sizes [14]. A *p* value < 0.05 was considered statistically significant. For each of the endpoints listed, two questions were addressed:

1. Do the values differ systematically over time? (e.g. main effect of time)
2. Do the changes over time differ systematically between treatments? (e.g. interaction effect between treatment and time)

All statistical analyses were performed using the statistics software R version 3.0.3 [15].

## Results

### Baseline characteristics

**Fig. 1:**
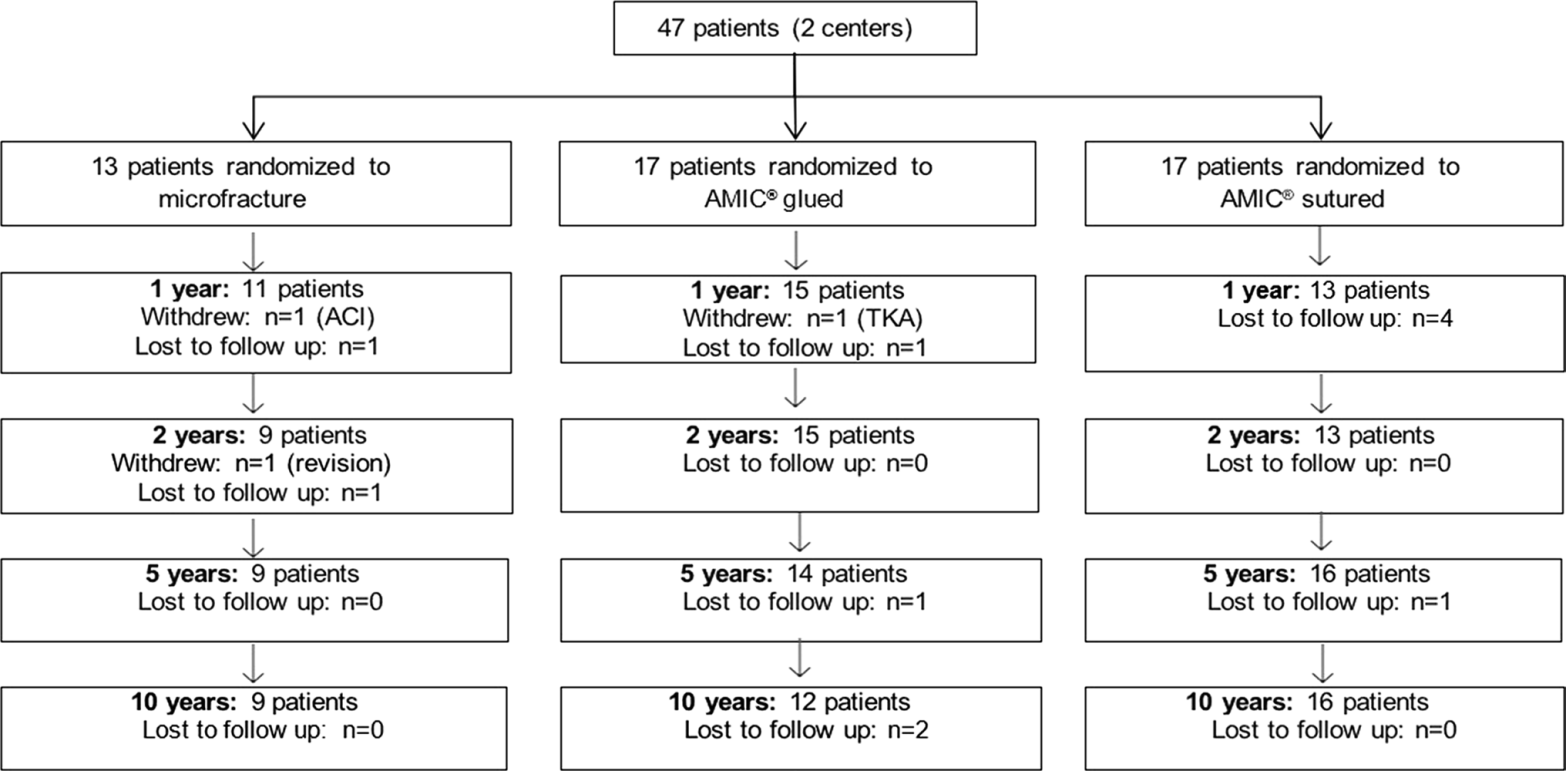
The CONSORT diagram for the patient allocation and follow-up. TKA: total knee arthroplasty; AMIC: autologous matrix-induced chondrogenesis; ACI: autologous chondrocyte implantation

There were 47 patients enrolled, and the 10-year follow-up included 37 patients as displayed in Fig. 1. The mean defect size after debridement was 3.6 cm^2^ (range 2.1–6.6 cm^2^). Demographic data for the patients are shown in Table 1, and a more detailed description can be found in our previous publication [9]. Demographic data did not differ between groups at baseline or at 10 years, except for lesion size. The demographic data are presented in Table 1.

**Table 1:** Demographic data of patients in each treatment group

	Lesion size (cm^2^)	BMI (kg/m^2^)	Age (years)	m/f(n)
MFx	2.9 ± 0.8	25.2 ± 2.1	39.9 ± 6.5	10/3
AMIC glued	3.9 ± 1.1	27.6 ± 4.1	38.7 ± 8.9	15/2
AMIC sutured	3.8 ± 1.9	27.4 ± 4.5	33.7 ± 11.5	12/5

^*^significance versus MFX: *p* = 0.01, MFX: microfracture; AMIC: autologous matrix-induced chondrogenesis; BMI: body mass index; m/f: male/female; n: number

## Clinical outcomes

### Modified Cincinnati score

**Fig. 2:**
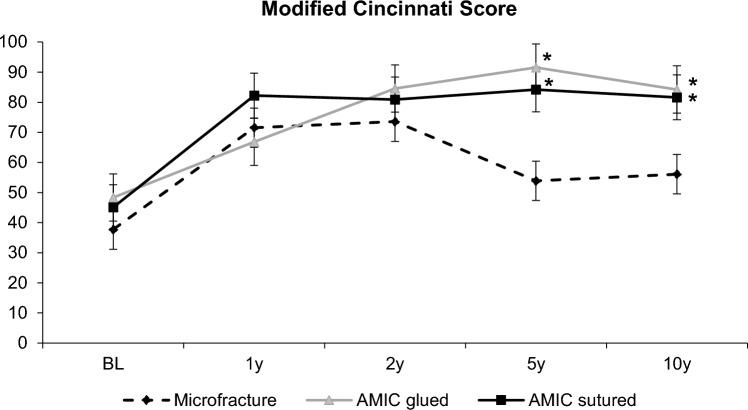
The mean scores over time for the MODIFIED Cincinnati knee score over time. (*significance over microfracture, *p* < 0.05). AMIC: autologous matrix-induced chondrogenesis; BL: baseline

For the Modified Cincinnati score, there was a significant overall change over time (*p* < 0.001). This time effect, however, differed significantly between the groups (Fig. 2). While at years 1 and 2, the changes from baseline were similar in all groups, a statistically significant difference between both AMIC^®^ groups and the MFx-treated patients was noted at years 5 and 10. The scores between the two AMIC^®^ groups (glued: 84.3 ± 17.1, sutured: 81.6 ± 21.2) and the MFx group (56.1 ± 18.6) showed a significant difference at 10 years, but no difference between the scores of either of the two AMIC^®^ fixation methods was detected.

### Pain

**Fig. 3:**
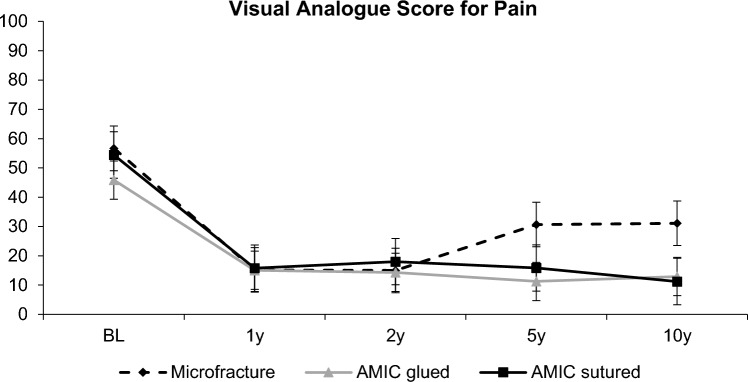
The data for the visual analogue scale for pain. AMIC: autologous matrix-induced chondrogenesis; BL: baseline

For the VAS pain, a similar result as for the Modified Cincinnati Knee Score could be observed. The mean baseline values were 57 ± 22, 46 ± 20 and 54 ± 19 for patients assigned to the MFx, AMIC^®^-glued and AMIC^®^-sutured groups, respectively. The changes in the pain score between groups are shown in Fig. 3. All patients showed a significant improvement in their pain scores up to 2 years (*p* < 0.001). After 5 years, both AMIC^®^-treated groups still reported very low pain, whereas pain increased non-significantly in the MFx group. Between year 5 and 10, pain was stable within the MFx group (30 ± 19 and 31 ± 20) and AMIC^®^-glued group (11 ± 20 and 12 ± 21) and slightly decreased in the AMIC^®^-sutured group (15 ± 22 and 11 ± 16). It should be noted that while there was an apparent divergence of the scores, this was not statistically significant.

### Clinical responders

**Fig. 4:**
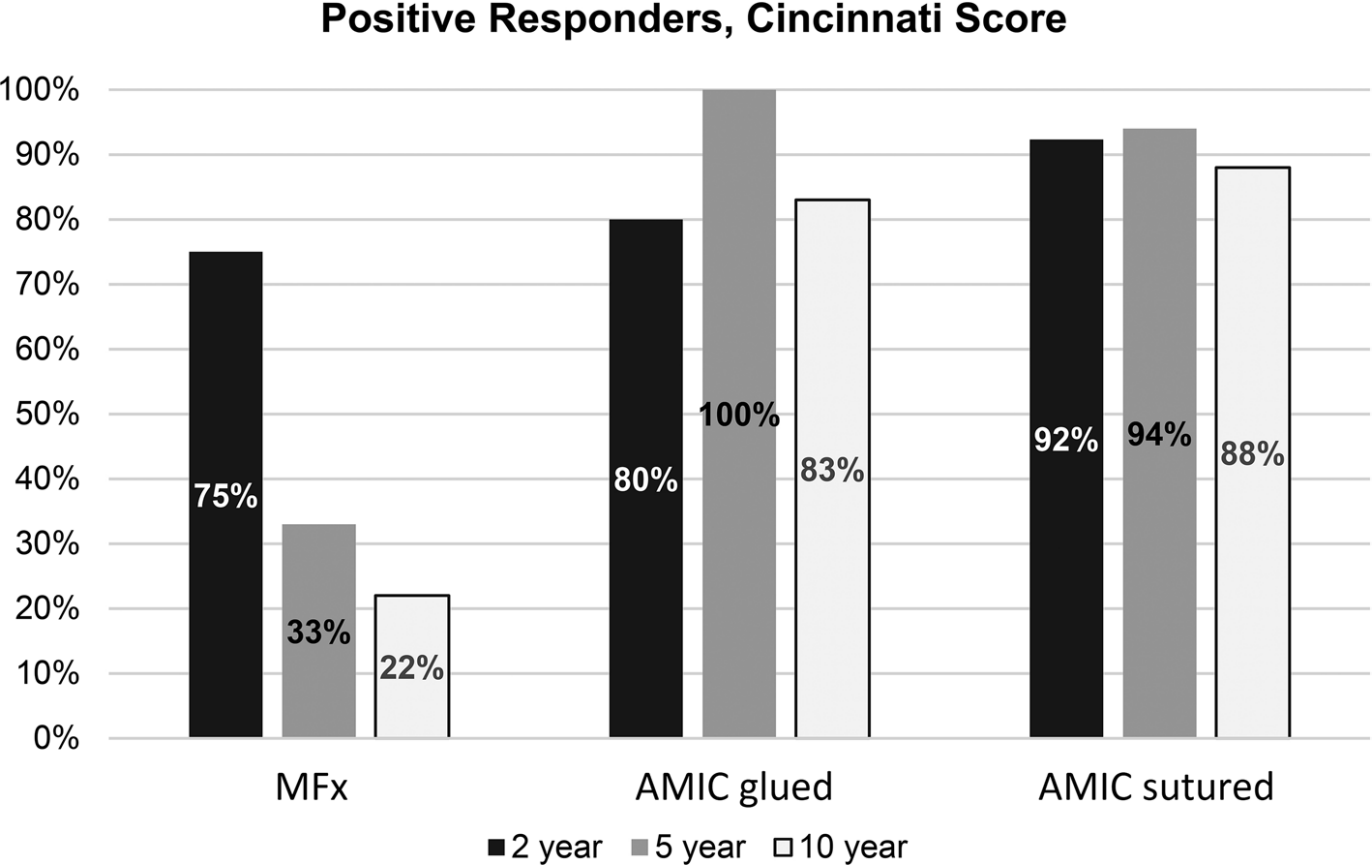
Percentage of positive responders (Cincinnati score > 65 pts.) in each cohort at different time points. MFX: microfracture; AMIC: autologous matrix-induced chondrogenesis

We considered the case a poor outcome if the Modified Cincinnati Knee Score was ≤ 65 points. This score was chosen as 62 has been cited to be the minimum Patient Acceptable Symptom State (PASS) score for the IKDC, while 70 has been cited as the PASS for the Lysholm [16]. Figure 4 depicts the proportion of patients in each treatment arm who exceeded this threshold at 2, 5 and 10 years after surgery. This reflects a responder rate of 22% for the MFx cohort at 10-year follow-up, while the AMIC^®^ cohorts showed a responder rate of 83% (glued) and 88% (sutured).

## Radiology

Of the 37 patients for whom we had 10-year data, MRIs were available for 32 (86%). The time between surgery and the date of the most recent MRI is shown in Table 2, with no significant difference between groups with respect to the date from surgery to the date of the MRI (*p* > 0.05).

**Table 2:** Time passed, in years, between the date of surgery and the date of the final MRI

	MFX	AMIC® glued	AMIC® sutured
Patients (n)	9	9	14
Mean	10.8	10.9	11.5
Median	11.1	10.9	11.7
Maximum	13.3	12.2	13.0
Minimum	7.8	9.4	9.5

AMIC: autologous matrix-induced chondrogenesis; MFx: microfracture; n: number

**Fig. 5:**
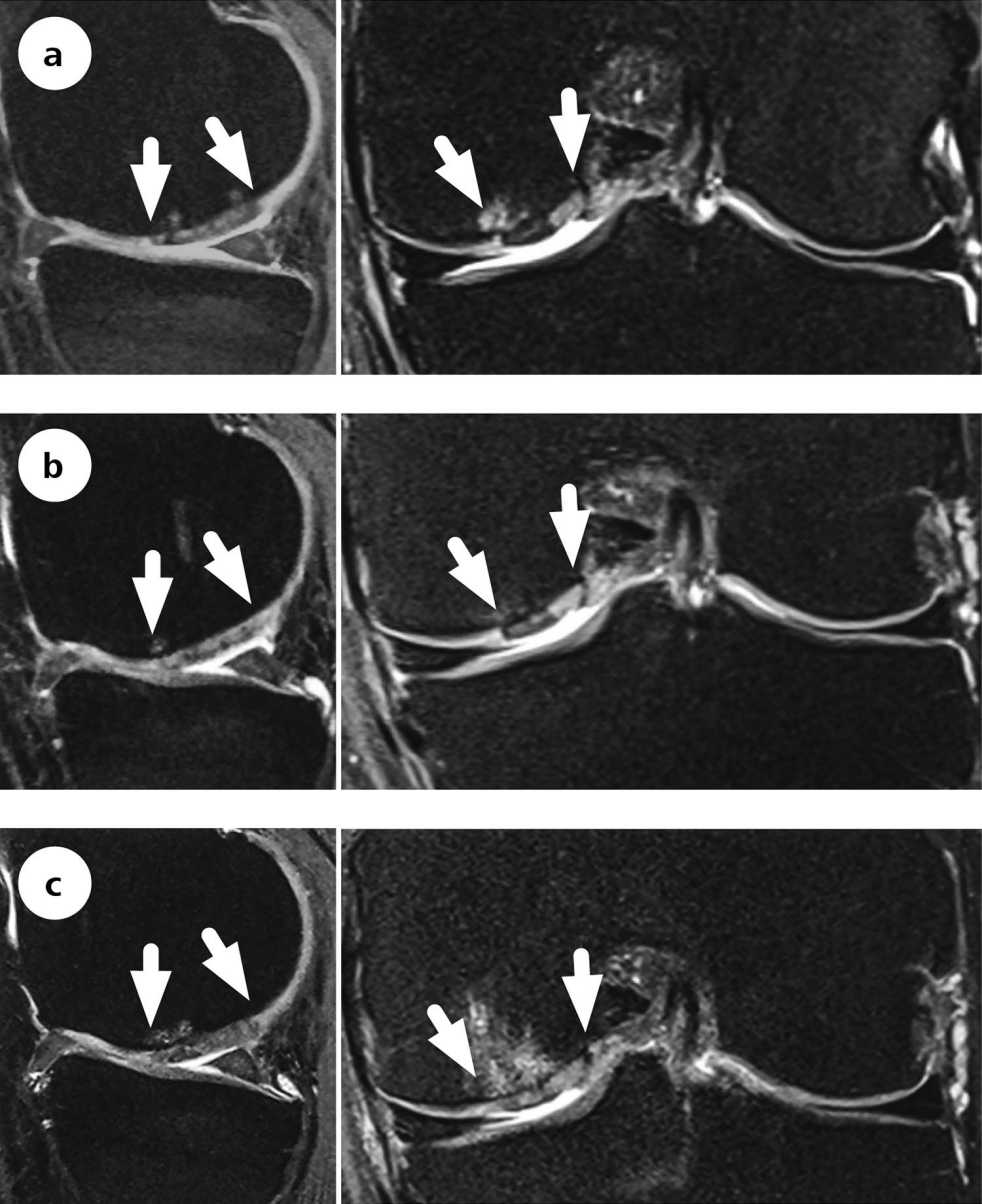
Representative MRI (1.5 T, t2PDw) with images of an AMIC^®^ glued at the medial femoral condyle at 2 years (**a**), 5 years (**b**) and 10 years (**c**). The Cincinnati score was 28/100/90/90 at baseline, 2, 5 and 10 years, respectively. The arrows indicate area of the surgical procedure

The mean overall MOCART scores at 10 years were 37.7 ± 29.3 (MFx), 34.4 ± 23.2 (AMIC glued^®^) and 31.0 ± 20.3 (AMIC^®^ sutured) points, respectively. A Kruskal–Wallis test showed no difference in the MOCART scores between the treatment groups (*p* = 0.879). The effusion was, at least in absolute terms, lower among the AMIC^®^-treated patients (data not shown). Regarding changes in subchondral bone, which would include osteophytes, comparable proportions of patients in each group showed evidence of changes in subchondral bone. Figure 5 shows a representative case MRI from one 46-year-old male patient treated with AMIC^®^ glued at the medial femoral condyle.

### Adverse events and failures

There were 2 patients in the MFx arm who underwent subsequent surgery (autologous chondrocyte implantation (ACI) at 12 months, a high tibial osteotomy (HTO) at 15 months), while 1 patient in the AMIC^®^-glued treatment arm converted to total knee arthroplasty (TKA) after 1 year. Between year 5 and year 10, no revision surgery was observed in any of the treatment groups. No serious AE related to the treatment was reported for any patient.

## Discussion

As a result of this PRCT, treating isolated cartilage defects in the knee with an AMIC^®^ procedure, either glued or sutured, demonstrated significantly higher Modified Cincinnati scores and lower VAS pain compared to patients having received a MFx alone at 10 years. Thus, our hypothesis was confirmed.

Over a 10-year follow-up for the surgical repair of focal chondral defects, our results showed that the initial outcomes were comparable between the treatment groups for the first 2 years, but diverged as time went on. Both AMIC^®^ cohorts maintained their improvement, whether measured via the VAS or the Modified Cincinnati score, while the MFx patients exhibited a worsening of these scores at the 5- and 10-year follow-ups. MOCART scores, however, were similar between all groups at the final follow-up. Additionally, with regard to safety, adverse events and failures were similar between the patient cohorts. Another result was a notable divergence between the treatment arms in relation to the clinical responder rate in the Modified Cincinnati score. Set at a minimum of 65 points, the proportion of MFx patients exceeding this threshold was notably smaller than seen in either of the AMIC^®^ groups (Fig. 4).

AMIC^®^ is a technique within the broader category of matrix-augmented bone marrow stimulation (mBMS). To our knowledge, this is the first PRCT comparing AMIC^®^ procedures versus conventional MFx over a 10-year period. Additionally, other studies have shown a sustained improvement in outcomes following the AMIC^®^ procedure. A recent analysis, based on an ongoing registry, has shown a stable clinical improvement up to 7 years post-operative [17]. Similarly, an RCT that had compared AMIC^®^ to AMIC^®^ and bone marrow aspirate concentrate (BMAC) demonstrated clinical improvements that were maintained up to 100 months status-post for both groups [18]. While both of those publications have shown sustainable outcomes over the longer term, the registry had no comparators, while the RCT compared the standard AMIC^®^ procedure to an AMIC^®^ procedure that added BMAC to the treatment site. Thus, a limitation is the lack of a comparison with different surgical procedures.

While microfracture is a simple, single-step arthroscopic procedure that has the longest clinical history, in terms of histological outcome, MFx manifested poorer tissue quality than other cartilage repair procedures [19]. In comparison with MFx, case–control studies have indicated that AMIC^®^ provides superior clinical outcomes and lower rates of failure or revision. Improved IKDC, Lysholm and pain scores in the AMIC^®^ group along with a lower rate of failure and a trend towards a lower rate of revision were noted when compared to MFx [20].

Aside from the AMIC^®^ procedure, there are a number of different scaffolds used in mBMS. Examples include cell-free type-1 collagen, as well as aragonite-based, chitosan, hyaluronan-based and a biomimetic nanostructure. Recently, a meta-analysis reported a significant improvement in outcomes for scaffold-associated repair procedures compared to microfracture at 2 years for focal cartilage defects in the knee of 1699 patients at 2 years [21]. Likewise, another meta-analysis noted significantly greater improvements in 744 patients with MFx + augmentation in the Lysholm score and post-operative MOCART scores compared with an isolated MFx treatment after 26.7 (12–60) months. Here, the mean chondral defect size ranged from 1.3 to 4.8 cm^2^ [19]. However, most of these procedures are quite limited with regard to clinical data, in contrast to the procedure that we perform which has a quantity of clinical data such that meta-analysis could be published [22].

Based on these data, the German Orthopaedic and Trauma Society (DGOU) guideline has stated that mBMS is standard of care treatment for focal chondral or osteochondral defects ranging from 1 to 4.5 cm^2^ [23]. In the context of mBMS, the use of the Chondro-Gide^®^ membrane in the AMIC^®^ procedure is based on sound clinical data, and the DGOU consensus statement rated it having the best clinical evidence [23]. AMIC^®^ and other mBMS procedures, however, are not the only procedures that have shown improved outcomes for chondral repair. The results from case series concerning ACI have shown a significant clinical improvement up to 25 years [24], while the clinical superiority of ACI relative to MFx at 5 years has also been published [25]. However, an RCT that compared collagen-covered ACI to AMIC^®^ noted no differences in outcomes after 2 years in patients with large defects (5 cm^2^) [26]. Another surgical option is osteochondral autologous cylinder transfer, for which long-term results are available, but some data suggest an increased risk of failure after 2 years, thus a caveat that these procedures might be more appropriate for smaller lesions [27].

Specific to the strength of clinical evidence and level 1 studies, there are relatively few RCTs that have compared surgical techniques for the repair of focal chondral defects. Similar to our data using MFx as a control group, an RCT that compared mosaicplasty to MFx reported better patient outcomes in the mosaicplasty cohort [28]. In contrast, an RCT that compared MFx to ACI noted that both groups improved their clinical scores in the short-, medium-, and long-term evaluations, with no significant difference at the long-term follow-up [29]. Most recently, a large RCT that evaluated outcome among a variety of procedures had stated that there was no evidence of a difference in Lysholm scores between ACI and alternative techniques, among which was AMIC^®^ [30].

With regard to objective measures of cartilage repair, while the clinical data indicated that patients treated using the AMIC^®^ surgical treatment had better long-term outcomes, our radiological data were inconclusive. While 3 of the 14 AMIC^®^-sutured patients showed complete filling of the treated site (21%), none of the MFx patients presented with this level of filling. With regard to joint effusion, 8/9 (88%) of the MFx patients for whom a 10-year MRI was available showed evidence of joint effusion, either mild or moderate, while this was 5/9 (55%) of the AMIC^®^-glued patients and 5/14 (36%) of the AMIC^®^-sutured patients. Our MRI data noted comparable rates of bony hypertrophy in the cohorts: AMIC^®^ 8/23 (35%) and MFx 3/9 (33%), which is comparable to what has been reported for mBMS or ACI [31]. Specific to MRI, recent research suggests that MOCART scores relate poorly to clinical outcomes [32]; therefore, the use of MOCART on assessment of surgical outcomes may have limited value.

We acknowledge our study limitations, the most obvious being low patient numbers at both enrolment and at 10 years. One of the challenges that face any PRCT is patient enrolment, and our study was no exception to this. However, in comparison with our 2-year and 5-year data [9], few patients were lost to follow-up between the 5-year and 10-year follow-up (Fig. 1). Another point worth noting in relation to the outcome scores is the lesion size. The defects for MFx (range 2–4.6 cm^2^, mean 2.9 cm^2^) were significantly smaller than defects in the AMIC^®^ groups (range 2.4–6.3 cm^2^, mean 3.9 cm^2^). While current recommendations limit MFx to defects ≤ 2 cm^2^ [23] and certainly no surgeon would today consider MFx for such lesions, it needs to be kept in mind that this guidance was developed several years after the surgeries in this study were performed. Indeed, the lesion size may impact the decrement in outcomes that we observed among the patients in the MFx cohort. Among the 3 groups in this study, there were 5 patients whose defects were greater than 5 cm^2^. When reviewing the case report forms, we noted that all of these patients had been randomized to an AMIC^®^ treatment, with 2 undergoing fixation via sutures and 3 whose repairs were secured using fibrin glue. Thus, the worst cases scenarios, with respect to defect size, had undergone an AMIC^®^ repair technique. Unfortunately, our sample size precludes a meaningful analysis, for example a linear regression, with regard to defect size and outcomes. The response rate is also an avenue for criticism. While there are established PASS values for IKDC and Lysholm scores, we approximated a PASS level for the Modified Cincinnati knee score, and while this is admittedly an arbitrary choice, it is close to both Lysholm and IKDC values [16].

Despite these limitations, our data provide evidence that the clinical outcomes for AMIC^®^, regardless of fixation, are superior to those of MFx alone for the treatment of focal, chondral lesions in the knee over 10 years and are consistent with our 5-year results [9].

## Conclusion

For the treatment of focal chondral lesions of the knee, the AMIC^®^ procedure, compared to microfracturing alone, maintains functional improvements and provides significantly better clinical outcomes at 10 years post-operatively.
